# Brain Injury and Neurodegeneration: Molecular, Functional, and Translational Approach 2.0

**DOI:** 10.3390/biomedicines12112586

**Published:** 2024-11-12

**Authors:** Pankaj Ahluwalia, Pankaj Gaur, Meenakshi Ahluwalia, Kumar Vaibhav

**Affiliations:** 1 Department of Pathology, Medical College of Georgia, Augusta University, Augusta, GA 30912, USA; pahluwalia@augusta.edu (P.A.);; 2 Lombardi Comprehensive Cancer Center, Georgetown University Medical Center, Washington, DC 20057, USA; pg684@georgetown.edu; 3 Georgia Cancer Center, Medical College of Georgia, Augusta University, Augusta, GA 30912, USA; 4 Brain Injury, Senescence and Translational Neuroscience Lab, Department of Neurosurgery, Medical College of Georgia, Augusta University, Augusta, GA 30912, USA

The brain is composed of different cells, such as neurons, glia, endothelial cells, etc., that perform specialized functions. Glial cells are key cells in the brain and are dominated by two major populations—astrocytes and microglia. These cells communicate with each other and other cells in the CNS, support the function of neurons, secrete cytokines as per immune and environmental clues, and maintain CNS homeostasis [1]. Astrocytes perform a wide range of functions in CNS homeostasis, and its reactivity to insult or pathological conditions is a heterogenous, dynamic, and context-dependent response [2]. The transforming growth factor-β (TGF-β) is a pleiotropic cytokine with a wide range of essential cellular functions, such as tissue homeostasis, and immune response and remodeling [3,4,5]. TGF-β has been identified as a key regulator of astrocyte reactivity, which can be manipulated to alter pathological and functional outcomes in brain diseases [6]. The CNS not only regulates the function of different organs of the body but is also affected by different organs and their functional components. The oral microbiota is the second most diverse microbiome population in the body. Through their systematic review, Giordano-Kelhoffer et al. highlight the importance of oral microbiomes in healthy and diseased conditions. Dysbiosis of the oral microbiota is clearly related with cardiovascular and neurodegenerative diseases [7,8]. The authors emphasized that lifestyle, diet, stress, alcohol, smoking, and health conditions actively modify the oral microbiome and may increase health complexities. Thus, maintaining a healthy lifestyle, physical activity, and a healthy diet aids in maintaining a balanced microbiome and thus a healthy body [9].

With the advancement of neuroscience research, we have come across various brain pathologies such as traumatic brain injury (TBI), hypoxic/hypobaric insults, hemorrhages, strokes, and neurological disorders such as Parkinson’s and Alzheimer’s diseases. Any insult to the brain (mild or severe) is multifactorial and initiates a cascade of inflammation, necrotic, and apoptotic pathways. It has long been known and established that brain insult or injury to the brain may lead to neurological disorders such as Alzheimer’s and Parkinson’s disease as time elapses, and genetic or environmental factors play significant roles in disease progression [10,11,12]. A substantial body of evidence has shown that oxidative stress, mitochondrial dysfunctions, protein aggregation and phosphorylation, excessive iron accumulation in the brain, and neuro-inflammation play a pivotal role in neurodegeneration and brain injuries [13,14,15]. The absence of a specific cure to restrain injury progression after an insult has prompted the scientific community to study the process behind the degenerative cascade and explore various therapeutic strategies.

Brain damage with clinical implications can be caused in two instances: either at birth or later in life. Any brain damage that transpires after birth, except for those caused by congenital disorders or degenerative diseases, is termed as acquired brain injury (ABI). Damage incurred from an ABI can last from days to a lifetime, depending on the severity of the injury, and has psychological, neurological, and physiological consequences [13]. Among all ABIs, TBI is a common cause of accidental long-term disability and brain pathologies in the adult population [16,17,18]. Adults surviving moderate and severe TBI often experience long-lasting neuropsychological, cognitive, and neuropsychiatric disorders with a prevalence rate ranging from 25 to 88% [19,20,21]. Chronic traumatic encephalopathy (CTE) is a chronic disease that develops after TBI and leads to long-term memory and cognitive impairment [22,23]. TBI-induced chronic pathologies and CTE show similarities with Alzheimer’s disease (AD) and other neurodegenerative diseases; therefore, a battery of diverse and innovative diagnosis tools is needed [23]. Advanced diagnostic tools such as diffusor tensor imaging, functional magnetic resonance imaging, positron emission tomography, and the fluid biomarkers t-tau, sTREM2, CCL11, NFL, and GFAP can be included to assess chronic effects post TBI [23]. Clinically, general cognitive tools such as the Mini Mental State Examination or more specific cognitive tests such as the Wisconsin Card Sorting Test and Trail Making Test could be implemented during the acute or chronic phase of TBI to assess the patient’s neuropsychological and cognitive function level [24]. Since the antemortem diagnosis of CTE is poor, future efforts toward clinical, radiological, metabolic, and molecular biomarker development are keys to improved diagnosis and prognosis [22].

Human activities and occupations in diverse environments have varying effects on the brain, particularly for individuals working in extreme conditions. It is critical to elucidate the genetic and environmental factors that influence its function, including a wide range of pathological conditions. Military personnel are exposed to different intensities of blasts and environmental conditions in war zones. Repetitive low-level blast exposure is one of the major occupational health concerns among US military service members and law enforcement and is associated with whole-blood dysregulated gene signatures mostly related to chronic inflammation and immune response, suggesting that these pathways may relate to the risk of lasting neurological symptoms following extended exposure to blast over a career [25]. In addition, environmental factors may also influence neuropathology and inflammation in military personnel. During the Gulf War, military personnel were exposed to paraoxon, an organophosphate. In a study by Freidin et al., the authors found that cognitive and behavioral deficits with neuroinflammation in the dentate gyrus were prominent in mice subjected to both mild TBI and paraoxon exposure [26]. In addition to external factors, endogenous factors such as hormones or previous stress episodes may affect TBI outcomes. The hypothalamic–pituitary–adrenal (HPA) axis is the main endocrine system that plays a critical role in late post-traumatic pathology, such as synaptic activity and neuroinflammation in the hippocampus. TBI leads to an alteration of cortisol levels due to altered HPA axis function. This glucocorticoid (cortisol in humans or corticosterone in animals) regulates two basic systems—glucose metabolism and immune response. The effect of glucocorticoid on the hippocampal neuronal population depends on its concentration, the duration of exposure, and the local cell population. Elevated levels of glucocorticoid may increase neuroinflammation and may be involved in chronic traumatic pathologies, such as epilepsy, depression, and cognitive impairment [27]. Thus, the excessive GCs/dysfunctional HPA axis may be responsible for neuroinflammation and distant hippocampal damage in many brain diseases. Besides direct brain injuries, substances of abuse can also affect brain health. Alcohol is one of the most common substances of abuse and can contribute to chronic brain pathologies. Skuja et al. investigated the substantia nigra section of alcoholic postmortem human brains and observed increased expression levels of collagen-IV, laminin-111, and fibronectin but decreased CD31^+^ vessels in the basement membrane of the blood–brain barrier (BBB) [28]. Waterpipe tobacco smoking (WPS) is prevalent in Asia and the Middle East but rapidly gaining popularity in other countries, especially among youths. A 6-month exposure study in mice revealed that WPS inhalation elevated the levels of DNA damage, lipid peroxidation, cytochrome C, IL-6, IL-1β, cleaved caspase-3, and nuclear factor-κB in the cerebellum. Thus, WPS may be associated with cerebellar inflammation, gliosis, oxidative stress, and apoptosis via NF-κB activation [29].

The brain consumes more oxygen and energy than any other organ in the human body, and its disruption can lead to widespread damage. Brain hypoxia because of stroke or TBI or any means is detrimental to motor function. The authors of a study examining primary motor cortex slices from 16–18-day-old infant rats demonstrated a high incidence of hypoxia-induced seizures associated with epileptiform motor behavior when exposed to post-natal hypoxia. This was linked to reversible depression in glutamatergic synaptic transmission and neuronal excitability mediated by adenosine acting on pre-synaptic A1 receptors to decrease glutamate release and the nitric oxide (NO)/cGMP postsynaptic pathway [30]. A better understanding of these molecular pathways would improve therapeutic efforts to treat this condition in humans.

Ischemic disease is the second most prevalent health problem globally with high mortality and morbidity. Temporary occlusion of the common carotid artery causes 25% of ischemic stroke cases [31]. The results of an experimental study examining male Wistar rats with temporary and permanent occlusion of either the common carotid artery or both showed an interesting effect on sciatic nerve motor-evoked potential (MEP) as a measure of the efferent transmission of the corticospinal tract. MEP amplitude decreased by 23.2% to 41.6% in 5 min and 10 min occlusion, respectively, while 5 min of arterial blood flow recovery stabilized MEP. While temporary occlusion did not evoke total and permanent inhibition in the activity of corticospinal tract neurons, bilateral occlusion was histologically more prominent and caused alteration in the sensory and motor areas controlling the forelimb [31]. Many factors can influence ischemic stroke outcomes and associated events. Arterial stiffness and vascular calcification increase with age and with the occurrence of ischemic events. The European Society of Cardiology recommends the use of polyunsaturated fatty acids (PUFAs) to reduce blood pressure, LDL, and inflammation and to increase NO in the vascular wall [32,33]. Levels of serum fatty acids are good indicators of the risk of ischemic stroke [34,35]. However, the effects of saturated and unsaturated fatty acids on stroke pathology remain inconsistent. Drozd et al. have assessed the impact of free fatty acids and their metabolites on non-dipping blood pressure and sleep apnea in 64 ischemic stroke patients [36]. Patients with preserved physiological dipping (DIP) showed higher scores on the Epworth sleepiness scale (ESS) with high levels of anti-inflammatory mediators from EPA and DHA acids; in comparison, 31 patients with the non-dipping phenomenon (NDIP) showed high levels of C18:3n6 gamma linoleic acid, indicating advanced inflammation events [36].

Over the past decade, several therapeutic approaches have emerged as promising strategies for addressing brain pathologies. The recombinant tissue plasminogen activator (rt-PA) is still a cornerstone of acute ischemic stroke treatment but is associated with bleeding complications [37,38]. In a study involving immortalized brain-derived endothelial cells (bEnd5) as a model of the BBB, rt-PA was found to cause cytotoxic and BBB damage, while the NLRP3-specific inhibitor MCC950 minimized this effect of rt-PA under ischemic conditions [39]. However, it is worth mentioning that ischemia and hypoxia are significant contributors to chronic neurodegeneration. Sharifulina et al. observed increased cytoplasmic levels of N- and C-terminal fragments of APP at 24 h post photothrombotic stroke, with an increase in co-immunoprecipitation with caveolin-1. The authors further reported that a caveolin inhibitor (Diadzein) enhanced Aβ synthesis from APP, while the γ-secretase inhibitor (DAPT) inhibited astrogliosis and reduced infarct volume after photothrombotic stroke [40].

The brain is the central organ of the nervous system, vital for daily functioning [41], and is among the organs most affected by toxicants and trauma, during aging, and its associated disorders. This Special Issue has provided a multidisciplinary platform for discussing the cellular functionality, brain pathology, and intervention of brain disorders. This issue emphasizes the pathological findings, the effect of environmental factors on the CNS and disease, and the mechanisms in the development of preventive and therapeutic strategies to limit brain injury and neurodegenerative disorders. In total, 15 articles were published as a part of this Special Issue, including 9 research articles, 5 reviews, and 1 systematic review, which complete the different aspects of this Special Issue and will provide a compelling read for the audience.
